# Research of Synergistic Substances on Tobacco Beetle [*Lasioderma serricorne* (Fabricius) (Coleoptera: Anobiidae)] Adults Attractants

**DOI:** 10.3389/fchem.2022.921113

**Published:** 2022-06-08

**Authors:** Yanling Ren, Tao Wang, Yingjie Jiang, Pengchao Chen, Jian Tang, Juan Wang, Daochao Jin, Jianjun Guo

**Affiliations:** ^1^ Guizhou Provincial Key Laboratory for Agricultural Pest Management of the Mountainous Region, Institute of Entomology, Scientific Observing and Experimental Station of Crop Pest in Guiyang, Ministry of Agriculture, Guizhou University, Guiyang, China; ^2^ Guizhou Light Industry Technical College, Guiyang, China

**Keywords:** chemical substances, plant essential oils, behavioral response, synergistic substances, tobacco beetle adults, laboratory simulation test

## Abstract

In this study, four kinds of chemical substances (2,3,5,6-tetramethylpyrazine, *β*-ionone, citronellal, and paeonol), three kinds of plant essential oils (tea tree essential oil, lavender essential oil, and myrrh essential oil), and their combinations were selected to explore their synergistic effects on tobacco beetle [*Lasioderma serricorne* (Fabricius) (Coleoptera: Anobiidae)] adults by the behavioral test and laboratory simulation test. Behavioral test results showed that some of the combinations revealed a synergistic effect on tobacco beetle adults, especially the sexual attractant +2,3,5,6-tetramethylpyrazine + *β*-ionone + citronellal + paeonol (SABCD, one portion of sexual attractant, and 1 mg/L synergistic substances) combination and the food attractant +2,3,5,6-tetramethylpyrazine + paeonol (FAD, 1 ml of food attractant and 1 mg/L synergistic substances) combination showed the best behavioral effect on tobacco beetle adults with average dwell times of 120.97 and 126.74 s, respectively, compared to those of other combinations. Meanwhile, SABCD had the highest selection rate [89.47%, about 1.5 times that of the sexual attractant (S)] on tobacco beetle adults compared with those of other combinations. In addition, laboratory simulation test results showed that the SABCD combination had the highest average selection rate (37.31%, about 2 times that of S) on tobacco beetle adults at 1 mg/L. However, our results showed that there was no significant difference in the indoor simulation results of food attractant synergistic substances. Our results will provide guidance for the development of new pesticides for tobacco beetle adults.

## Introduction

Insects are divided into pests and beneficial insects. Beneficial insects need to be developed and utilized, and pests need to be controlled. At present, the control methods for pests are mainly physical control methods (Lü et al., 2022; Sang et al., 2022), biological control methods (Cheng et al., 2022; Hu et al., 2022), and chemical control methods (Bi et al., 2022; Chi et al., 2022). The commonly used pesticides in chemical control methods cause pest resistance and environmental pollution (McCaffery, 1998; Saglam et al., 2015; Tang et al., 2022), and some pesticides are banned in many countries currently (Scholler et al., 2018). However, the products of sex attractants (Ikeda et al., 1980; Cao et al., 2020) and food attractants (Cai et al., 2018; Lu et al., 2020) rely on insect sex pheromones (Miao, 1989; Aziz et al., 1992) and plant essential oils or volatiles with attractive effects on insects (Light et al., 2001; Oliver and Mannion., 2001; Cai et al., 2018), respectively. Therefore, humans and the environment are very good and is a very popular chemical control method at present. Sex attractants rely on the principle that insects release sex pheromones to attract the opposite sex to come to mate and reproduce offspring (Miao, 1989; Li et al., 2012). Te sex attractants have been developed since the 1960s (Butenandt and Hecker, 1961; Regnier and Law, 1968) and have achieved good results in many pest control and monitoring applications, such as the *Cydia pomonella* sex attractant (Xiang et al., 2021), the *Spodoptera frugiperda* sex attractant (Huang et al., 2022), and the *Conogethes punctiferalis* sex attractant agent (Chen et al., 2022). Food attractants rely on the principle that herbivorous insects need to feed on plants to obtain the ability or rely on plants to synthesize some scarce substances (Knolhoff and Heckel, 2014; Cai et al., 2018), thereby attracting pests to achieve the goal of pest control. The food attractants of fruit fly pests (Shelly, 2010; Shelly et al., 2014; Gregg et al., 2016), Lepidoptera pests (Sutherland et al., 1977; Knight et al., 2015; Gregg et al., 2016), thrips pests (Niassy et al., 2011; Broughton and Harrison, 2012; Davidson et al., 2015; Mfuti et al., 2016), and beetle pests (Jackson et al., 2005; Ranger et al., 2011; Chen et al., 2014) are greatly developed and applied.

Attractants are widely used in the monitoring and control of pests. But most of the single attractants can only target male insects (Li et al., 2012) and the effect of a single attractant is not as good as the combination of different compounds (Lampman and Metcalf, 1988; Landolt et al., 2014; Knight et al., 2015). Therefore, the attractants composed of a large number of chemical substances and have attracting effects on insects are more meaningful for pest control. For example, *Bactrocera dorsalis* (Hendel) food attractants are composed of eugenol, matrix, and toxicant (Jang et al., 2013), and the bollworm food attractant contains 2-phenylethanol, phenylacetaldehyde, and volatiles found primarily in leaves (Gregg et al., 2010). The food attractants of *Diabrotica* spp. are composed of 1,2,4-trimethoxybenzene, indole, and *trans*-cinnamaldehyde (Lampman and Metcalf, 1987), and the M99 and G04-7 attractants of *Monochamus alternatus* Hope are composed of α-pinene, *β*-pinene, acetaldehyde, and acetone (Chen et al., 2014). In addition, FJ-MA-02, PE, PA, A-3, and SC-1 attractants, etc., of *M. alternatus* are composed of different chemical substances (Chen et al., 2014).

Tobacco beetle [*Lasioderma serricorne* (Fabricius) (Coleoptera: Anobiidae)] is a worldwide storage pest that harms tobacco (Linnie, 1994; Lü and Ma, 2015; Edde, 2019). Its sex pheromone (4*S*,6*S*,7*S*)-4,6-dimethyl-7-hydroxynonan-3-one (serricomine) was first isolated from female tobacco beetles in the 1980s (Chuman et al., 1979) and is still the main component of most sex pheromone traps (Papadopoulou and Buchelos, 2002; Athanassiou et al., 2018). In addition, the sex pheromone had 4,6-dimethyl-7-hydroxy-3-nonanon and 2,6-diethyl-3,5-dimethyl-2,3-dihydropyran (Mao et al., 1992), which are also slowly applied in the control of tobacco beetle. Studies on the synergistic substances of tobacco beetle sex attractants include the following: Du (2006) studied the synergistic effects of Hangbaiju, Coriander, Gongju, and Brazilian flue-cured tobacco on sexual attractants, and the combined effect of Hangbaiju and pheromone was the most effective (; Xiong (2008) studied the synergistic effect of the combination of *Angelica sinensis*, citronella, fennel, alfalfa, and green tea on sex attractants; and Guarino et al. (2022) studied the synergistic effect of *β*-ionone on tobacco beetle sex pheromone, and the best-combined effect was about 1.5 times that of sex attractant alone. However, apart from plant essential oils and plant volatiles about tobacco beetle food attractants, there are currently no specific attractant products (Lu and Liu, 2016; Cao et al., 2019; Guarino et al., 2021).

Therefore, in this study, using the chemical substances and plant essential oils with good attracting effects on tobacco beetle adults reported in the previous studies (Lü and Liu, 2016; Cao et al., 2019; Guarino et al., 2021; Zhong et al., 2021; Ren et al., 2022) as research materials, we explore their synergistic effects of four kinds of chemical substances (2,3,5,6-tetramethylpyrazine, *β*-ionone, citronellal, and paeonol), three kinds of plant essential oils (tea tree essential oil, lavender essential oil, and myrrh essential oil), and their combinations on tobacco beetle adults by the behavioral test and laboratory simulation test.

## Material and Methods

### Test Insects

Tobacco beetle adults were obtained from the Institute of Entomology of Guizhou University and reared at the Guizhou Engineering Research Center for Mountain Featured Fruits and Products of Guizhou Light Industry and Technical College. The feeding feed was composed of corn residue, yeast powder, and tobacco leaf powder (15: 1: 0.75). The feeding equipment was an artificial intelligence climate box. The rearing conditions were as follows: photoperiod of 16L: 8D, relative humidity of 60 ± 5%, and temperature of 28 ± 1°C. About 12 h before the experiment, tobacco beetle adults that had emerged within a week were selected and placed in a 100 ml transparent packing box.

### Test Materials

Sexual attractant (S), food attractant (F), and sticky cardboard were purchased from Henan LoveTree Technology Development Co. Ltd. (Henan, China). 2,3,5,6-Tetramethylpyrazine (A) was purchased from Shanghai Aladdin Biochemical Technology Co, Ltd. (Shanghai, China). *β*-Ionone (B) was purchased from Shanghai Macklin Biochemical Co., Ltd. (Shanghai, China). Citronellal (C) and paeonol (D) were purchased from Shanghai Macklin Biochemical Co., Ltd. (Shanghai, China). Tea tree essential oil (E), lavender essential oil (G), and myrrh essential oil (H) were purchased from Beijing Maosi Trading Company (Beijing, China). The combinations of the test materials are shown in Table 1.

**TABLE 1:** The combinations of the test materials.

Full name	Abbreviation	Full name	Abbreviation
Sexual attractant	S	Food attractant	F
Sexual attractant +2,3,5,6-tetramethylpyrazine	SA	Food attractant +2,3,5,6-tetramethylpyrazine	FA
Sexual attractant +*β*-ionone	SB	Food attractant + *β*-ionone	FB
Sexual attractant + citronellal	SC	Food attractant + citronellal	FC
Sexual attractant + paeonol	SD	Food attractant + paeonol	FD
Sexual attractant +2,3,5,6-tetramethylpyrazine + *β*-ionone	SAB	Food attractant +2,3,5,6-tetramethylpyrazine + *β*-ionone	FAB
Sexual attractant +2,3,5,6-tetramethylpyrazine + citronellal	SAC	Food attractant +2,3,5,6-tetramethylpyrazine + citronellal	FAC
Sexual attractant +2,3,5,6-tetramethylpyrazine + paeonol	SAD	Food attractant +2,3,5,6-tetramethylpyrazine + paeonol	FAD
Sexual attractant + *β*-ionone + citronellal	SBC	Food attractant + *β*-ionone + citronellal	FBC
Sexual attractant + *β*-ionone + paeonol	SBD	Food attractant + *β*-ionone + paeonol	FBD
Sexual attractant + citronellal + paeonol	SCD	Food attractant + citronellal + paeonol	FCD
Sexual attractant +2,3,5,6-tetramethylpyrazine + *β*-ionone + citronellal	SABC	Food attractant +2,3,5,6-tetramethylpyrazine + *β*-ionone + citronellal	FABC
Sexual attractant +2,3,5,6-tetramethylpyrazine + *β*-ionone + paeonol	SABD	Food attractant +2,3,5,6-tetramethylpyrazine + *β*-ionone + paeonol	FABD
Sexual attractant + *β*-ionone + citronellal + paeonol	SBCD	Food attractant + *β*-ionone + citronellal + paeonol	FBCD
Sexual attractant +2,3,5,6-tetramethylpyrazine + *β*-ionone + citronellal + paeonol	SABCD	Food attractant +2,3,5,6-tetramethylpyrazine + *β*-ionone + citronellal + paeonol	FABCD
Sexual attractant + tea tree essential oil	SE	Food attractant + tea tree essential oil	FE
Sexual attractant + lavender essential oil	SG	Food attractant + lavender essential oil	FG
Sexual attractant + myrrh essential oil	SH	Food attractant + myrrh essential oil	FH
Sexual attractant + tea tree essential oil + lavender essential oil	SEG	Food attractant + tea tree essential oil + lavender essential oil	FEG
Sexual attractant + tea tree essential oil + myrrh essential oil	SEH	Food attractant + tea tree essential oil + myrrh essential oil	FEH
Sexual attractant + lavender essential oil + myrrh essential oil	SGH	Food attractant + lavender essential oil + myrrh essential oil	FGH
Sexual attractant + tea tree essential oil + lavender essential oil + myrrh essential oil	SEGH	Food attractant + tea tree essential oil + lavender essential oil + myrrh essential oil	FEGH
Sexual attractant +2,3,5,6-tetramethylpyrazine + *β*-ionone + citronellal + paeonol + tea tree essential oil + lavender essential oil + myrrh essential oil	SABCDGH	Food attractant +2,3,5,6-tetramethylpyrazine + *β*-ionone + citronellal + paeonol + tea tree essential oil + lavender essential oil + myrrh essential oil	FADEG

### Behavioral Test of Attractants

About 50 μL of 1 mg/L (chemical substances) or 1 μL/L (plant essential oils) of the substance (dilute with absolute ethanol) is taken and dripped onto a 2 cm × 4 cm filter paper strip. The amount of sex attractant and food attractant in the experiment is 1 portion and 1 ml, respectively, for each trap required by the product instructions. The filter paper strip and attractant were put into the test source bottle and the filter paper strip was changed every 5 times. The gas source bottle and subsequent equipment from side to side were swapped every 20 times to reduce any risk of affecting the results of the experiment. After the behavioral test for each combination, the gas source bottle and all subsequent equipment pieces were first cleaned with alcohol 2–3 times, then cleaned with ultrapure water 2–3 times, and finally, dried at 70°C for 30 min.

**FIGURE 1:**
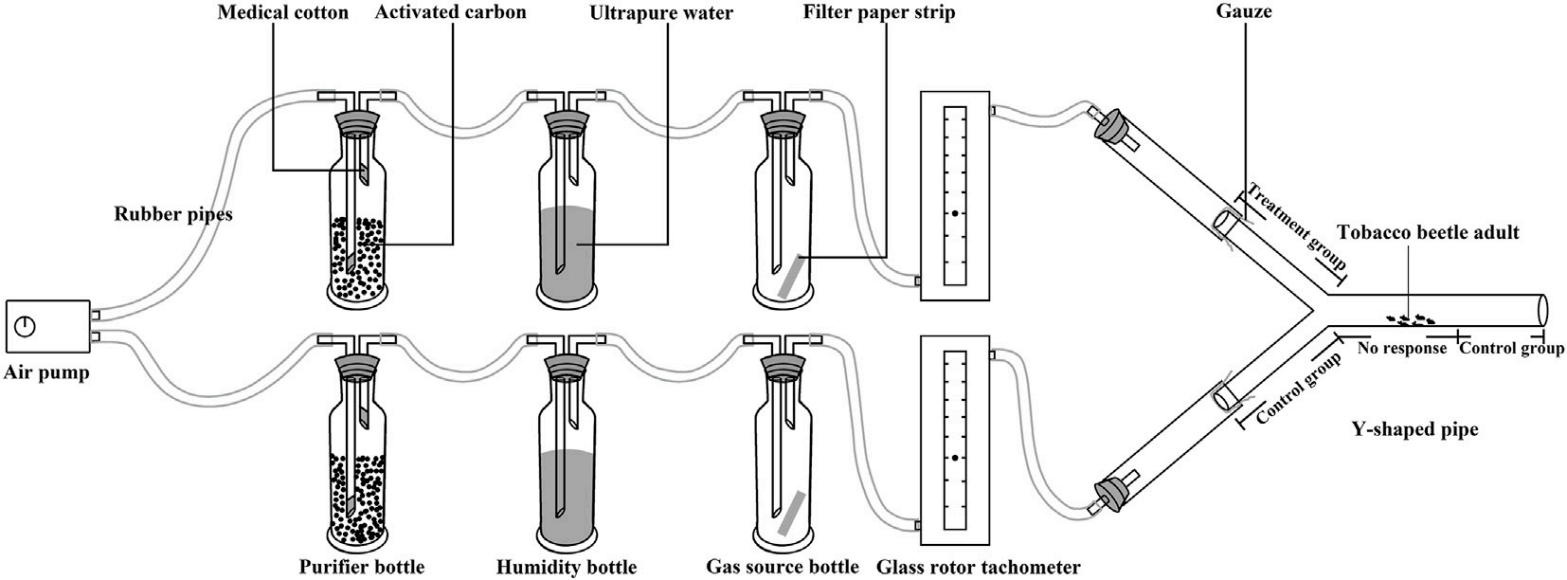
The diagram for the behavioral test on tobacco beetle adults.

The research method of the synergistic effect of four chemical substances and three plant essential oils, as well as their combinations on tobacco beetle sexual attractant and food attractant, is based on the study reported by Guarino et al. (2021), and the diagram for the behavioral test on tobacco beetle adults is shown in Figure 1. The temperature of the test room was controlled at 28 ± 1°C by an air conditioner. An air pump (Aco-5505, Guangdong Haili Group Co., Ltd., all instruments are connected by rubber pipes with inner and outer diameters of 6 and 9 mm, respectively) to generate airflow. The airflow passes through 1,000 ml bottles filled with activated carbon to purify impurities and then passes through a 250 ml humidity bottle containing ultrapure water to increase air humidity. The airflow rate was adjusted through the airflow meter (flow rate: 500 ml/min); the airflow passed through the 250 ml gas source bottle to carry the smell and then entered the Y-shaped pipe (stem: 20 cm; arms: 15 cm at a 140 angle; stem internal diameter: 5.0 cm, arms internal diameter: 3.5 cm, placed in an evenly lit area). After 5 min of ventilation, one tobacco beetle adult was placed in the middle of the main stem (3–5 cm away from the Y-pipe connection).

The time when the tobacco beetle adult entered the experimental group (test arm) and the control group (the tobacco beetle entered the blank arm and the stem 5 cm away from the connection) was recorded and continued to observe for 300 s. The dwell time is calculated by the following formula:

$$\text{Average dwell time }\left(\text{s}\right)=\frac{\text{Test or control group insect active time}}{\text{Test group insects numbers}+\text{Control group insects numbers}},$$

Meanwhile, after the laboratory behavioral test, the tobacco beetle was placed in a 2 ml centrifuge tube, and then male and female identification was carried out using a stereoscopic microscope (SZ680, Chongqing Auto Optical Instrument Co., Ltd.). The abdomen was slightly squeezed with tweezers to expose the genitals. The genitals are simple in shape for females and are complex for males. Only sex attractant synergistic substance studies are conducted to identify males and females.

### Laboratory Simulation Test

The blank rubber attractant core (Henan LoveTree Technology Development Co. Ltd., Henan, China) was immersed in absolute ethanol for 24 h, dried for 12 h, and then soaked in 20 ml of 0, 1, 10, and 20 mg/L (chemical substances) or 1 μL/L (plant essential oils) reagents (chemicals and plant essential oils) for 24 h. Finally, it was taken out and placed on a sticky insect board during the experiment.

**FIGURE 2:**
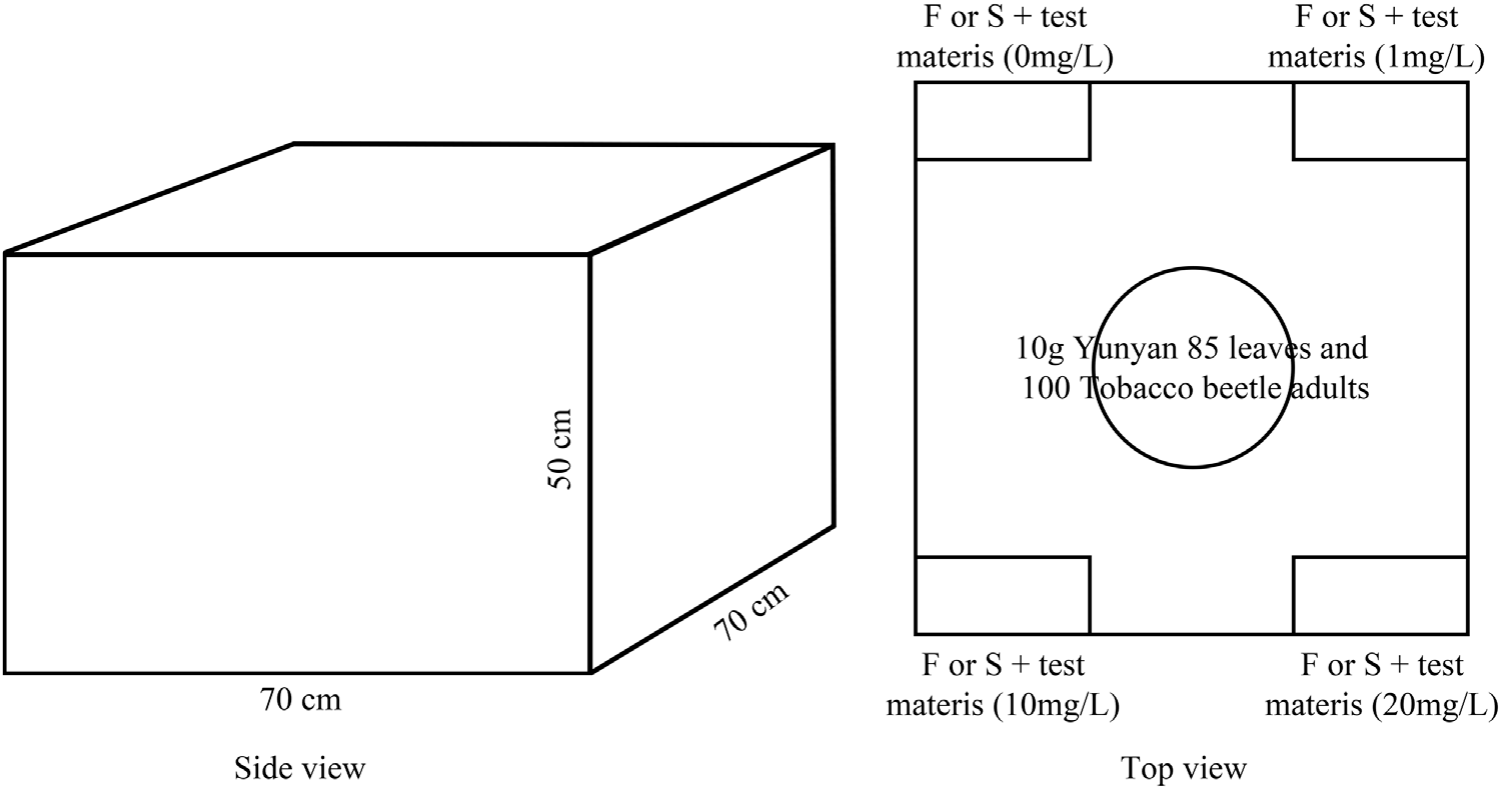
The diagram for the laboratory simulation test on tobacco beetle adults.

After the laboratory behavior test, the synergistic substances with the longest average dwell time were selected to be used in the laboratory simulation test. The experimental method referred to the research of Li et al. (2020) and the diagram for the laboratory simulation test on tobacco beetle adults is shown in Figure 2. The test materials were divided into four concentration gradients of 0, 1, 10, and 20 mg/L or μL/L, plus one sexual attractant or 1 ml of food attractant to form four test gas sources. The temperature of the test room was controlled at 28 ± 1°C by an air conditioner, and 10 g of Yunyan 85 leaves was placed in the middle of the wooden box with the length, width, and height of 70, 70, and 50 cm, respectively, to simulate the tobacco warehouse environment (the top of the wooden box was covered with gauze to prevent the escape of tobacco armor and ensure ventilation). The sticky insect boards were put down on the four corners of the wooden box, the test source was placed in the center of the sticky insect board, and 100 tobacco beetle adults were put in the bottom center point of the wooden box. After 24 h, the sticky insect board was collected, and the number of insects was recorded. After the laboratory simulation test, the wooden box was moved to the outdoor for 24 h ventilation. A total of 12 repetitions were performed, with each repetition moving the sticky insect boards clockwise. The selection rate is calculated by the following formula:

$$\text{Selection rate }\left(\text{\%}\right)=\frac{\text{Test or control group insects numbers}}{\text{Test group insects numbers}+\text{Control group insects numbers}}\times 100,$$

### Statistical Analysis

Basic processing of data was performed using Microsoft Office Excel (version 2016). LSD test was conducted in SPSS (version 22), and the difference level was *p* < 0.05.

## Results

### Behavioral Test of Sexual Attractants

**FIGURE 3:**
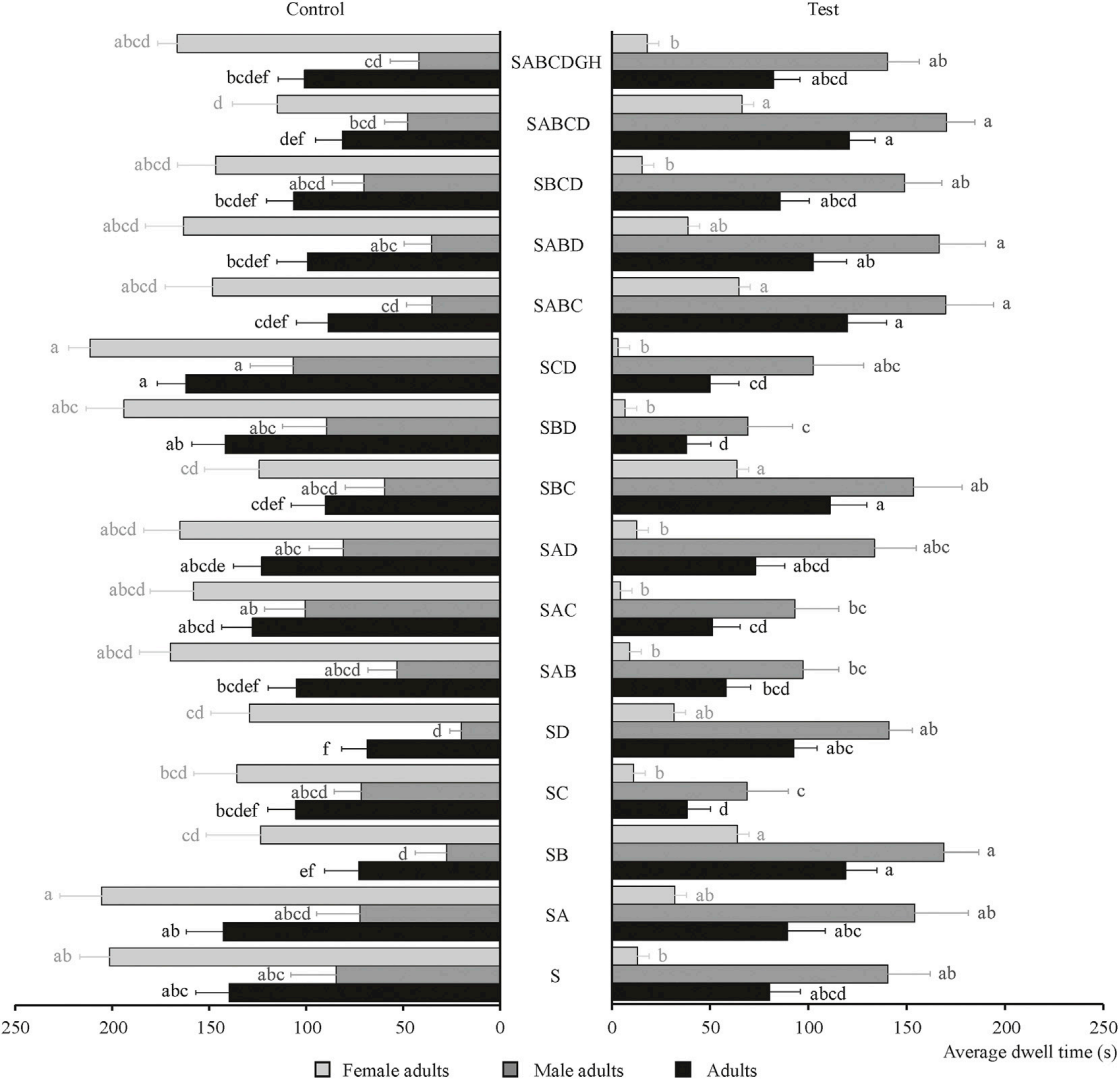
An average dwell time of male adults, female adults, and adults of tobacco beetle in the combination of chemical substances and sexual attractants. Different lowercase letters in the figure represent the average dwell time of male adults, female adults, and adults of tobacco beetles with a significant difference at *p* < 0.05 in different combinations. Error bars shown in the figure represent mean ± SE.

The behavioral effects of four chemical substances and their combinations on the tobacco beetle sexual attractant were determined and shown in Figure 3. As shown in Figure 3, the chemical substance that had the best behavioral effect on sexual attractants is B; its average dwell time is 119.05 s, which is about 1.5 times that of S (80.33 s). Also, SABCD revealed a synergistic effect on tobacco beetle adults with the longest average dwell time of 120.97 s, which is about 1.5 times that of S. Meanwhile, the average dwell times of the SABCD combination for male and female adults were 170.25 and 66.22 s, respectively, which were 1.2 and 5.1 times than those of S (140.53 and 13.06 s, respectively), demonstrating that the SABCD combination also revealed a synergistic effect on male and female adults. Other substance combinations plus sexual attractants with synergistic effects are SA, SB, SD, SBC, SABC, SABD, and SBCD, while it is puzzling that the average dwell times of the combination of SC, SAB, SAC, SAD, SBD, and SCD were lower than those of S. The tobacco beetle adults remained in the experimental group of SD, SB, SABCD, SABC, SBC, SABD, etc., more than the corresponding control group.

**FIGURE 4:**
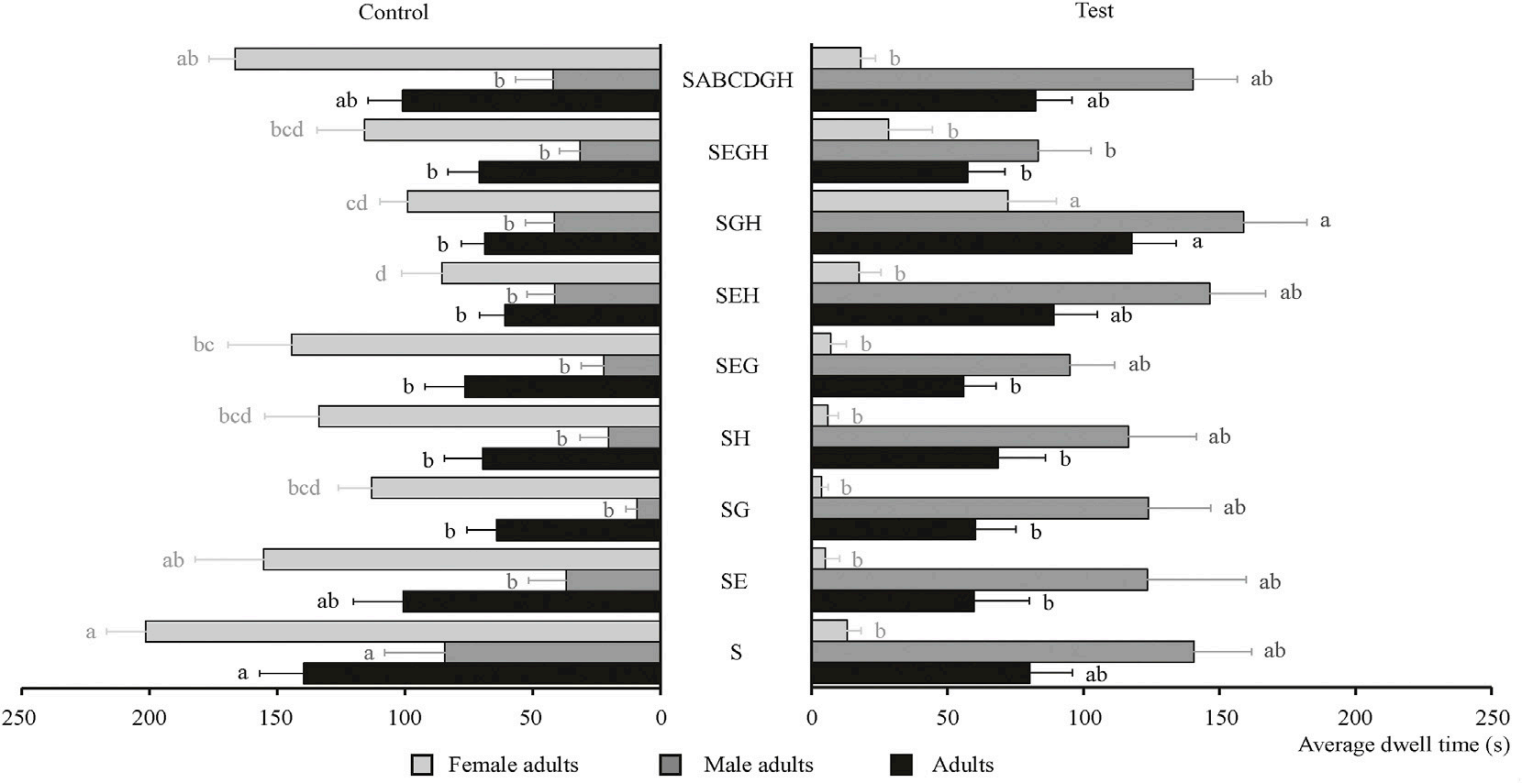
Average dwell time of male adults, female adults, and adults of tobacco beetle in the combination of plant essential oils and sexual attractants. Different lowercase letters in the figure represent the average dwell time of male adults, female adults, and adults of tobacco beetle with a significant difference at *p* < 0.05 in different combinations. Error bars shown in the figure represent mean ± SE.

The behavioral effects of three plant essential oils and their combinations on the tobacco beetle food attractant were determined and shown in Figure 4. As shown in Figure 4, each of the three plant essential oils on the sexual attractant had no obvious synergistic effect on male adults, female adults, and adults of tobacco beetle compared with S. The longest average dwell time was with SGH combination, and its average dwell time (117.84 s) was about 1.5 times than that of S (80.33 s), demonstrating that SGH combination had a synergistic effect on tobacco beetle adults. The average dwell time (158.85 s) of male adults in the SGH combination was 1.1 times that of S (140.53 s); meanwhile, the average dwell time (72.28 s) of female adults in the SGH combination was about 5.5 times that of S (13.06 s), also demonstrating that the SGH combination had a synergistic effect on male and female adults. Figure 3 also shows that the synergistic effects of SEH and SGH combinations are positive, and other plant essential oil combinations are all negative. The tobacco beetle adults remained in the experimental groups of SEH and SGH more than the corresponding control group, and SGH is about 1.7 times.

### Behavioral Test of Food Attractants

**FIGURE 5:**
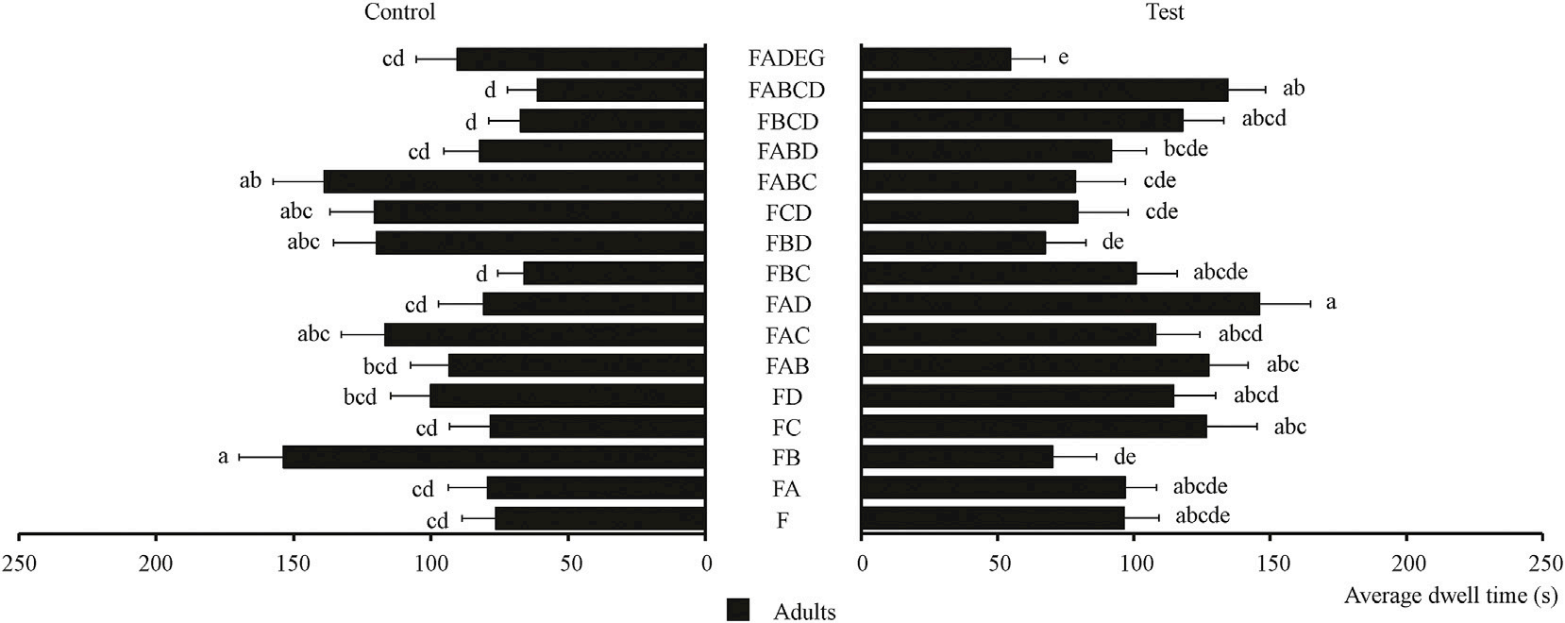
Average dwell time of tobacco beetle adults in the combination of chemical substances and food attractants. Different lowercase letters in the figure represent the average dwell time of tobacco beetle adults with a significant difference at *p* < 0.05 in different combinations. Error bars shown in the figure represent mean ± SE.

The behavioral effect of four chemical substances and their combinations on food attractants were determined and the results are shown in Figure 5. Figure 5 shows that the chemical substance that had the best behavioral effect on tobacco beetle adults is citronellal [average dwell time is 126.74 s, 1.3 times than F (80.33 s)]. Meanwhile, Figure 5 also shows that the FAD combination showed the best synergistic effect on tobacco beetle adults with the longest average dwell time of 146.15 s, which was about 1.5 times that of F (96.45 s). Among these combinations, nine combinations (FA, FC, FD, FAB, FAC, FAD, FBC, FBCD, and FABCD) had synergistic effects on tobacco beetle adults, while six combinations (FB, FBD, FCD, FABC, and FABD) had no synergistic effect on tobacco beetle adults.

**FIGURE 6:**
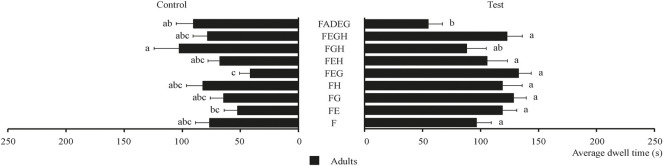
Average dwell time of tobacco beetle adults in the combination of plant essential oils and food attractants. Different lowercase letters in the figure represent the average dwell time of tobacco beetle adults with a significant difference at *p* < 0.05 in different combinations. Error bars shown in the figure represent mean ± SE.

The behavioral effect of three plant essential oils and their combinations on food attractants was determined and the results are shown in Figure 6. As shown in Figure 6, all the combinations had no obvious synergistic effect on tobacco beetle adults compared with F. FEG had the highest average dwell time (132.75 s), which is about 1.4 times that of F (96.45 s).

### Analysis of Selection Rate

From the characteristics of the sticky insect board, in reality, once the insects choose to enter the range of the sticky insect board, they cannot be re-selected. Therefore, according to the data record, we also analyzed the selection rate of S plus on different chemical substances, plant essential oils, and their combinations, respectively. As shown in Table 2, SABCD had the highest selection rate (89.47%) on tobacco beetle adults compared with those of S and other combinations. Meanwhile, the male selection rates of SD, SABCD, SEG, and SABCDGH combinations reached 100%, which was even better than those of S (84.21%) and other combinations. In addition, SC had the best female selection rate (94.74%) than those of S (35.29%) and other combinations.

**TABLE 2:** The selection rates of sexual attractant (S) plus different chemical substances, plant essential oils, and their combinations.

Combination Abbreviations	Adults selection rate (%)		Male selection rate (%)		Female selection rate (%)	
	Test	Control	Test	Control	Test	Control
S	61.11	88.89	84.21	78.95	35.29	100.00
SA	52.94	70.59	87.50	50.00	22.22	88.89
SB	78.95	47.37	95.00	25.00	61.11	72.22
SC	33.33	88.89	58.82	82.35	94.74	63.16
SD	77.78	61.11	100.00	40.00	50.00	87.50
SAB	55.56	77.78	80.00	60.00	25.00	100.00
SAC	38.89	88.89	63.16	78.95	11.76	100.00
SAD	61.11	94.44	88.89	88.89	33.33	100.00
SBC	66.67	61.11	89.47	52.63	41.18	70.59
SBD	33.33	94.44	44.44	88.89	22.22	100.00
SCD	38.89	88.89	64.71	76.47	15.79	100.00
SABC	63.16	52.63	95.00	30.00	27.78	77.78
SABD	65.00	65.00	90.00	35.00	40.00	95.00
SBCD	68.43	78.95	90.00	70.00	44.44	88.89
SABCD	89.47	78.95	100.00	63.16	78.95	94.74
SE	30.77	69.23	58.33	41.67	23.53	94.12
SG	47.06	64.71	87.50	25.00	11.11	100.00
SH	60.00	60.00	94.12	29.41	15.38	100.00
SEG	61.11	58.33	100.00	30.00	12.50	93.75
SHE	69.44	66.67	95.00	65.00	37.50	68.75
SGH	73.68	73.68	80.00	55.00	66.67	94.44
SEGH	56.25	75.00	82.35	64.71	26.67	86.67
SABCDGH	73.68	68.42	100.00	40.00	44.44	100.00

Meanwhile, the selection rate of food attractants plus different chemical substances, plant essential oils, and their combinations, respectively, were also analyzed, and the results are shown in Table 3. As shown in Table 3, the adult selection rate of FE reached 100.00%, which was even better than F (85.00%) and other combinations. In addition, the average adult selection rate of the combination of synergistic substances and food attractants was higher than that of the combination of synergistic substances and sexual attractants (75.10% > 58.98%).

**TABLE 3:** The selection rate of the food attractant (F) plus different chemical substances, plant essential oils, and their combinations.

Combination abbreviations	Adults’ selection rate (%)	
	Test	Control
F	85.00	70.00
FA	80.00	75.00
FB	50.00	85.00
FC	73.68	68.42
FD	70.00	75.00
FAB	90.00	65.00
FAC	75.00	75.00
FAD	80.00	50.00
FBC	80.00	70.00
FBD	50.00	70.00
FCD	40.00	75.00
FABC	47.37	78.95
FABD	76.47	88.24
FBCD	81.08	59.46
FABCD	95.00	70.00
FE	100.00	55.56
FG	94.74	68.42
FH	84.21	57.89
FEG	95.00	50.00
FEH	75.00	65.00
FGH	61.76	41.18
FEGH	90.00	70.00
FADEG	52.94	70.59

### Laboratory Simulation Test

After the laboratory behavior test, the synergistic substances of SABCD and FAD were selected to be used in the laboratory simulation test, and the results are shown in Table 4. Table 4 reveals that the average response rate of the FAD combination is 41.5%, which is equal to that of the SABCD combination (39.5%). Meanwhile, the SABCD combination had the highest average selection rate (37.31%) on tobacco beetle adults at 1 mg/L, which was 2.14 times the average selection rate at 0 mg/L, and with the increase of the concentration of the synergistic substance, the average selection rate has a tendency to decrease. In addition, Table 4 also shows that the average selection rate of FAD had no significant change with the increase in the concentration of the synergistic substances.

**TABLE 4:** The average response rate and selection rate of SABCD and FAD combinations on tobacco beetle adults.

Combination abbreviations	Average response rate (±SE) (%)	Concentration of synergistic substances (mg/L)	Average selection rate (±SE) (%)
SABCD	39.50 (±3.95)^a^	0	17.41 (±2.07)^c^
		1	37.31 (±1.91)^a^
		10	23.47 (±1.55)^b^
		20	21.81 (±1.63)^bc^
FAD	41.50 (±3.10)^a^	0	21.71 (±3.42)^a^
		1	32.28 (±3.40)^a^
		10	20.01 (±4.21)^a^
		20	26.00 (±4.78)^a^

The average response rate uses an independent-sample *t-*test. Different lowercase letters in the figure represent the average respones rate or average selection rate of tobacco beetle adults with a significant difference at *p* < 0.05 in different combinations.

## Discussion

In our study, the synergy of *β*-ionone and other substances was better (about 1.5–2 times) than that of single *β*-ionone for food/sex attractants; however, the synergistic effect of Hangbaiju on the tobacco nail attractant (Du, 2006), the synergistic effect of angelica + citronella on the tobacco nail attractant (Xiong, 2008), the synergistic effect of *β*-ionone on the tobacco nail attractant (Guarino et al., 2022), and their synergistic effect was not more than 1.5 times. But, judging from the research on attractants of other insects, the multiplier was far more than 2 times, and the number of *Mythimna separata* trapped by the combination of ethyl benzoate and armyworm sex pheromone was about 4–5 times that of armyworm sex pheromone (Yang et al., 2015). The combination of (*Z*)-8-dodecenyl acetate, (*E*)-8-dodecenyl acetate, and codlemone (95: 4: 10) was found to trap *Grapholita molesta* adults about 5–6 times more than the commercial sex attractants with the combination of (*Z*)-8-dodecenyl acetate, (*E*)- 8-dodecenyl acetate, and (*Z*)-8-dodecenol (95:4:1) (Liu et al., 2021).

In addition, some combinations had a very good lure effect on tobacco beetles, but after combining these best combinations, the effect was not as good as when it was not combined or the effect does not increase significantly. Similar results were also reported in other studies. Ataide et al. (2020) studied the behavioral response of different essential oils to tobacco beetle adults, but the synergistic effect of eucalyptol and euggenol combination was not better than a single one of them. Fornari et al. (2013) found that the average number of *Lobiopa insularis* was caught after 7 days of exposure to different food attractants, and the mixture of ripe strawberries and combination (dairy cattle feed, granulated sugar, and water) was not as large as their individual catches. Sathiyaseelan et al. (2022) found that rice bran and rice flour alone were more effective in attracting insects such as *Sitotroga cerealella*, *Rhyzopertha dominica*, *Tribolium* spp., *Sitophilus oryzae*, and *Oryzaephilus surinamensis* than their combinations. Therefore, it is very necessary to study the compounding of substances that have a good attraction to tobacco beetles.

## Conclusion

In conclusion, the synergistic effects of four kinds of chemical substances, three kinds of plant essential oils, and their combinations on tobacco beetle adults were determined by the behavioral test and laboratory simulation test. Behavioral test results showed that the SABCD combination showed the best synergistic effect and selection rate on tobacco beetle adults. Meanwhile, laboratory simulation test results showed that the SABCD combination had the highest average selection rate on tobacco beetle adults at 1 mg/L, demonstrating that the SABCD combination could be further used for the tobacco beetle adults' control. However, our results showed that there was no significant difference in the indoor simulation results of food attractant synergistic substances, providing guidance for controlling tobacco beetle adults.

## Data Availability

The original contributions presented in the study are included in the article/Supplementary Material, further inquiries can be directed to the corresponding authors.
